# Circulação do conhecimento, uma proposta incontornável

**DOI:** 10.1590/S0104-59702026000100039

**Published:** 2026-10-12

**Authors:** Ricardo dos Santos Batista, Gabriela dos Reis Sampaio

**Affiliations:** i Professor, Programa de Pós-graduação em História, Universidade Federal da Bahia. Salvador – BA – Brasil. batista.ufba@hotmail.com; ii Professora, Programa de Pós-graduação em História, Universidade Federal da Bahia. Salvador – BA – Brasil. grsampaio@hotmail.com

**Figure:**
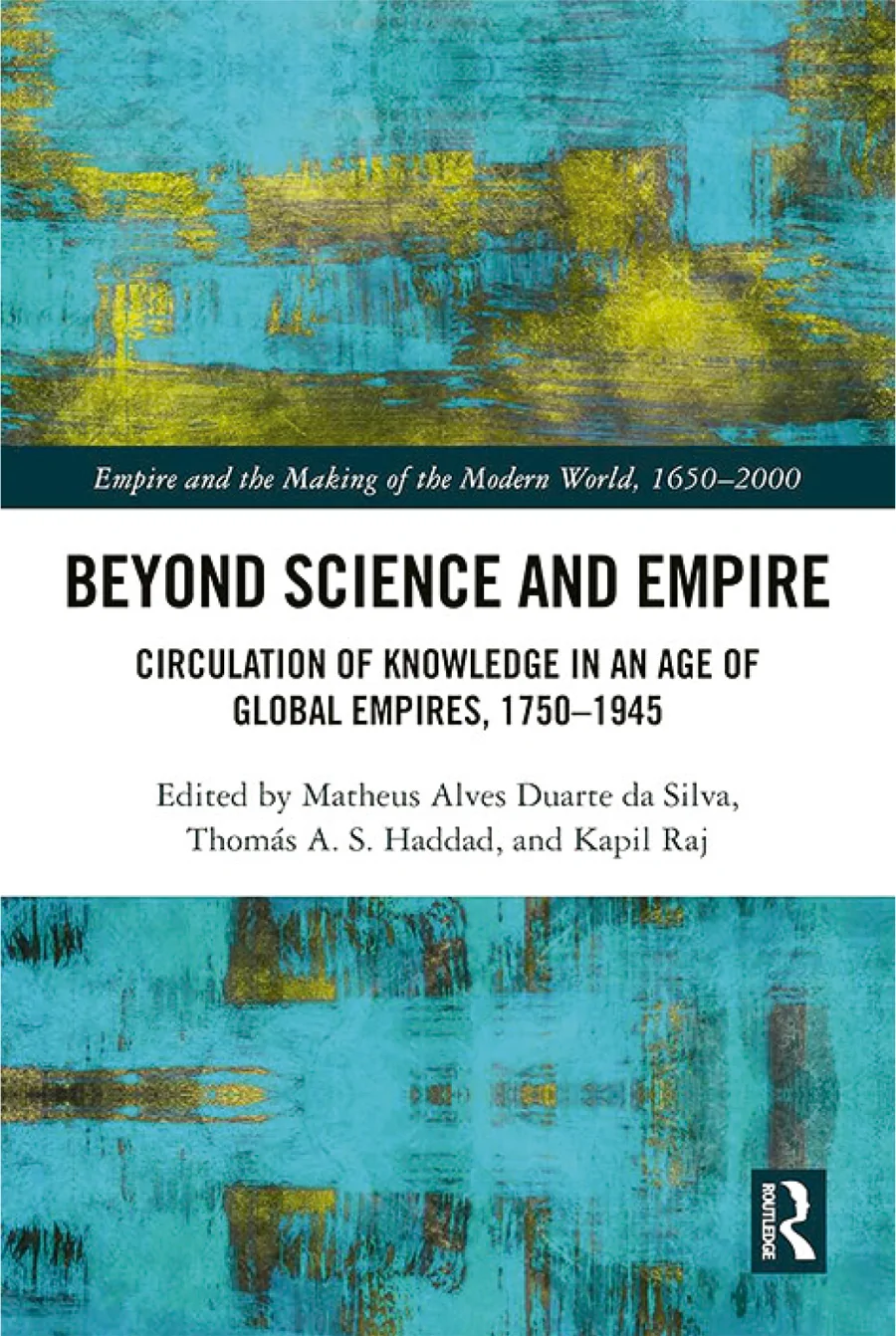

Nos últimos anos, observa-se uma preocupação, especialmente nos países do chamado Sul Global, em acionar ferramentas teórico-metodológicas que se desprendam do eurocentrismo que, por muito tempo, dominou as “formas de conhecer” em países que foram colonizados. O pós-colonial foi visto como umo momento posterior à emancipação das sociedades exploradas pelos colonizadores e seus imperialismos, a exemplo do que ocorreu na África, na Ásia e na América Latina e, ao mesmo tempo, como produtor de contribuições teóricas oriundas dos estudos literários e culturais que, a partir dos anos 1980, ganharam evidência em universidades dos EUA e da Inglaterra. É também uma corrente de pensamento que se opõe às relações de colonização, colonialismo e colonialidade e possui referências importantes como Alberto Memmi, Aimé Césaire e Frantz Fanon. No estágio atual da produção historiográfica, diferentes intelectuais promovem desdobramentos desse pensamento e avançam na crítica ao conceito de ciência como uma produção exclusiva da Europa, associando-se intimamente à proposta de uma história global desenvolvida por abordagens como as histórias conectadas, a história comparada, a história transnacional, entre outras (Santos Jr., Sochaczewski, 2017).

Nesse sentido, parcerias estabelecidas entre pesquisadores de centros de estudo em história das ciências no mundo têm produzido reflexões importantes. Um exemplo dos resultados dessas conexões é a coletânea *Beyond science and empire: circulation of knowledge in an age of global empires, 1750-1945*, publicada pela Routledge, em 2024, e organizada por Matheus Duarte da Silva, pesquisador no Departamento de Saúde Global e Medicina Social do King’s College, Londres. Thomás Augusto Santoro Haddad, professor de história das ciências na Escola de Artes, Ciências e Humanidades da Universidade de São Paulo, Brasil; e Kapil Raj, pesquisador do Centre Alexandre Koyré, da École des Hautes Études en Sciences Sociales, França.

A coletânea enfatiza a circulação do conhecimento em zonas imperiais, consideradas por muito tempo o “resto” do mundo, na esteira de outros trabalhos – a exemplo de Raj (2007) – que questionaram amplamente o modelo proposto por George Basalla, historiador norte-americano da ciência para o qual a expansão colonial e imperial europeia abriu caminho para a difusão da ciência moderna pelo mundo. Avança, assim, em relação aos estudos da chamada “virada imperial” e de produções latino-americanas que, ao refletir sobre “ciência e império” e sobre as complexidades de uma possível “transferência” da ciência moderna da Europa para a América Latina, respectivamente, denunciaram o conhecimento científico como elemento facilitador dos processos ocidentais de dominação, mas não problematizaram a própria concepção de ciência europeia.

O objetivo maior do conjunto de dez capítulos organizados por Silva, Haddad e Raj em duas partes – “Knowledge production on imperial landscapes” e “Knowledge production at imperial crossroads” – é responder a críticas direcionadas ao conceito de circulação do conhecimento. Algumas delas argumentam que a circulação do conhecimento seria capaz de invisibilizar a violência inerente à vida cotidiana nos impérios e ignorar a destruição de vidas e formas de conhecimento indígenas; outras acreditam que o foco em redes internacionais e fluxos globais leva a história da ciência a subestimar ou ignorar as estruturas de poder que conformam e moldam as interações globais; e, por fim, também se afirma que o termo “circulação” tem sido utilizado estrategicamente como substituto de conceitos anteriores, a exemplo de difusão, transferência e disseminação, sem problematizá-los ou apresentar qualquer inovação que contribua para uma análise crítica no debate.

A circulação do conhecimento, conforme proposta por Raj (2007), não se circunscreve ao ato de mover ou transitar, mas às relações estabelecidas entre agentes que negociam, resistem e disputam poder. A circulação observada em diferentes tradições científicas, portanto, não apenas move o conhecimento produzido, mas o modifica, tanto em forma quanto em conteúdo. Isso é percebido em estudos de caso detalhados dos processos pelos quais as habilidades, práticas e instrumentos associados são essenciais, em detrimento das grandes narrativas ou dos relatos abrangentes, o que auxilia na percepção da natureza contingente, negociada e situada dos acontecimentos históricos.

É justamente por meio das análises de casos que dão ênfase aos chamados “indivíduos mediadores”, personagens que interagem nas “zonas de contato” entre diferentes culturas e tradições científicas, que os autores da coletânea se lançam ao desafio e abrem novas possibilidades frente a reflexões já realizadas com foco nesses elementos (Raj, 2007; Pratt, 2008; Batista, Porto, Lopes, 2023). Assim, evidenciam experiências como a de Joseph Banks, que mostra como, em finais do século XVIII, navios britânicos que transportavam espécies botânicas não eram apenas jardins-botânicos flutuantes, mas locais de produção de conhecimento científico que envolviam agentes como capitães e jardineiros.

A segunda parte do livro é digna de destaque. De acordo com os organizadores, seu principal objetivo é apresentar o que aconteceu com o conhecimento quando atores – humanos ou não humanos – cruzaram fronteiras físicas, políticas e científicas e, devido aos seus movimentos, conectaram diferentes localidades. Esse processo envolveu o contato entre lugares físicos – como a Alfândega Marítima Imperial, criada pela dinastia Qing no século XIX para mediar o comércio entre o império chinês, EUA e países europeus; e fronteiras imperiais construídas metaforicamente e “borradas pela circulação de objetos”, como se observa nos trabalhos de Saint-Hilaire. Em seus escritos, o naturalista viajante ampliava as dimensões simbólicas do império brasileiro que se formava, e seu próprio prestígio, no Velho Mundo.

*Beyond science and empire: circulation of knowledge in an age of global empires, 1750-1945* é um ótimo exemplo de como a história global pode ser escrita sem desprezar as importantes contribuições que os procedimentos metodológicos da micro-história agregaram ao conhecimento histórico. A *microstoria* italiana, é bom lembrar, trouxe em seus debates a preocupação com a relação do estudo de casos, da microanálise, com a realidade mais ampla, em um jogo de escalas entre o micro e o macro sem hierarquias fixas, em uma conexão dinâmica, o que poderia trazer, a partir do pequeno, a perspectiva mais abrangente para o entendimento dos contextos (Levi, 2003).

Ao recuperar desafios metodológicos de diferentes correntes histográficas e apresentar análises centradas em ampla pesquisa e cuidadoso detalhamento de casos específicos, em vasta localização geográfica, a obra contribui para consolidar uma proposta incontornável, marcada pela utilização do conceito de circulação do conhecimento. Diante das transformações no contexto geopolítico internacional que apresentam o protagonismo de países anteriormente considerados “periféricos” ou subdesenvolvidos, e apesar das críticas ao termo, a circulação configura um instrumento poderoso na construção de interpretações que diminuem as assimetrias entre conhecimentos produzidos na metrópole e nos impérios e que reconhece o papel desempenhado por agentes locais, os quais foram por tanto tempo invisibilizados na historiografia que reflete sobre aspectos internacionais.

Na historiografia contemporânea, baseada em pesquisa ampla de tantos e tão variados casos pelo planeta, bem como em metodologia sofisticada, não é possível mais reafirmar que a ciência nos países marcados pelo colonialismo seria uma cópia pálida do que se produziu na metrópole, um mero legado recebido da Europa pelo “restante do mundo”, tampouco entender a relação entre impérios e ciência como simples e direta. Para compreender melhor essas ideias, recomendamos a leitura do livro.

## Data Availability

Não estão em repositório.
